# Carbon-Doped TiO_2_ Nanofiltration Membranes Prepared by Interfacial Reaction of Glycerol with TiCl_4_ Vapor

**DOI:** 10.3390/membranes14110233

**Published:** 2024-11-07

**Authors:** Wenjing Zhang, Jiangzhou Luo, Honglei Ling, Lei Huang, Song Xue

**Affiliations:** Tianjin Key Laboratory of Organic Solar Cells and Photochemical Conversion, School of Chemistry & Chemical Engineering, Tianjin University of Technology, Tianjin 300384, China; zhangwj@stud.tjut.edu.cn (W.Z.); linghl@email.tjut.edu.cn (H.L.); huanglei@tjut.edu.cn (L.H.)

**Keywords:** ceramic membrane, organic solvent nanofiltration, TiO_2_, glycerol, stability

## Abstract

In the pursuit of developing advanced nanofiltration membranes with high permeation flux for organic solvents, a TiO_2_ nanofilm was synthesized via a vapor–liquid interfacial reaction on a flat-sheet α-Al_2_O_3_ ceramic support. This process involves the reaction of glycerol, an organic precursor with a structure featuring 1,2-diol and 1,3-diol groups, with TiCl_4_ vapor to form organometallic hybrid films. Subsequent calcination in air at 250 °C transforms these hybrid films into carbon-doped titanium oxide nanofilms. The unique structure of glycerol plays a crucial role in determining the properties of the resulting nanopores, which exhibit high solvent permeance and effective solute rejection. The synthesized carbon-doped TiO_2_ nanofiltration membranes demonstrated impressive performance, achieving a pure methanol permeability as high as 90.9 L·m^−2^·h ^−1^·bar^−1^. Moreover, these membranes exhibited a rejection rate of 93.2% for Congo Red in a methanol solution, underscoring their efficacy in separating solutes from solvents. The rigidity of the nanopores within these nanofilms, when supported on ceramic materials, confers high chemical stability even in the presence of polar solvents. This robustness makes the carbon-doped TiO_2_ nanofilms suitable for applications in the purification and recovery of organic solvents.

## 1. Introduction

Traditional separation processes like distillation and evaporation frequently encounter high energy consumption as a result of the heat of vaporization, which accounts for over 40% of total expenses in the chemical and pharmaceutical industries [1,2]. Efficient separation processes are crucial for reducing costs and achieving economic and environmental sustainability [3]. Membrane-based separation offers advantages over traditional processes, including no need for a phase change, a smaller footprint, and higher energy efficiency [4]. Compared to water treatment and gas separation, processing organic solvents through membranes remains relatively underdeveloped largely due to the difficulty of stabilizing membrane materials in hash solvents [5]. In the past few decades, many industrial procedures in the fields of pharmaceuticals and specialty chemicals have demanded the isolation of solutes from organic solvents, consequently promoting the advancement of organic solvent nanofiltration (OSN) [6,7]. OSN has attracted numerous researchers and has spurred the development of polymer membranes with improved resistance toward organic solvents [8]. Cross-linked polyimide (PI) membranes have been proven to possess good chemical resistance in a wide range of organic solvents, such as acetone, toluene, and methanol [9,10]. However, one of the major obstacles is low solvent permeability, which limits the application of the OSN technique [11]. Thus, new materials are desirable to achieve high solvent permeability with energy-efficient separation.

In recent years, novel emerging microporous materials, such as polymers of intrinsic microporosity (PIMs) [12,13,14], covalent organic frameworks (COFs) [15,16,17], metal organic frameworks (MOFs) [18,19], and metallic oxide [20,21], have garnered much attention for fabricating OSN membranes. Polymeric nanofiltration membranes provide benefits such as flexibility, a straightforward preparation process, and comparatively low cost [22]. Metallic oxide nanofiltration membranes exhibit enhanced chemical resilience, thermal endurance, and mechanical robustness. These attributes make them suitable for applications under extreme operating conditions [23]. Ceramic membranes, typically prepared from Al_2_O_3_, ZrO_2_, and TiO_2_, using sol–gel processes involving alkoxide precursors followed by drying and sintering [24], generally comprise a macroporous support, a mesoporous intermediate layer, and a microporous selective layer. The selective layer effectively separates small organic molecules, while the support and intermediate layers give the desired robustness and stability [21]. Ensuring the meticulous regulation of the deposition procedure is crucial for the fabrication of superior ceramic nanofiltration membranes. In the conventional method, a gel is meticulously synthesized from a colloidal dispersion through the incorporation of organic modifiers, which serve to modulate the hydrolysis and condensation reactions involving alkoxide species [25]. Additionally, membrane thickness for the selective layer cannot be precisely controlled by the sol–gel method, severely limiting the preparation of ultrathin, high-flux nanofiltration membranes. Moreover, pore sizes of metal oxides’ nanofiltration films prepared by the sol–gel process are not easy to precisely control at about 1 nm, particularly for stable ceramic membranes [26].

Molecular layer deposition (MLD) has attracted significant attention as a suitable technique for depositing organic–inorganic hybrid films [27,28]. MLD exhibits self-limiting growth behavior where film growth occurs solely through the chemical adsorption of metal precursors and multifunctional organic alcohols [29]. A highly controllable preparation of TiO_2_ nanofiltration membranes with about 1 nm pores was demonstrated using MLD [30]. The thin hybrid films were formed on the surfaces of an anodic aluminum oxide (AAO) support using TiCl_4_ and ethylene glycol (EG). Since TiCl_4_ reacts smoothly with a layer of EG molecules to form a cross-linked species (TiOCH_2_CH_2_OTi), surface reactions are carried out alternately to deposit organic–inorganic hybrid materials coated on the substrate. These hybrid materials were transformed into porous TiO_2_ nanofilms through calcination. Low-temperature calcination resulted in an amorphous TiO_2_ nanofilm that could serve as a membrane for nanofiltration, whereas high-temperature calcination yielded a crystalline TiO_2_ nanofilm that could act as an ultrafiltration membrane [31]. In addition to calcination temperature, organic precursor sizes also influenced the pore sizes of the nanofiltration membrane. Smaller precursors make the pore size smaller, allowing only small-size molecules to pass through, and vice versa. Therefore, pore sizes of nanofiltration can be controlled by adjusting organic precursors and calcination temperatures. The MLD technique employed for nanofilm deposition is characterized as an interfacial reaction mechanism, which signifies the creation of metal oxide materials with precisely controlled pore sizes that facilitate molecular sieving capabilities.

Very recently, M. Xu et al. reported high-flux nanoporous carbon-doped metal oxide (CDTO) nanofilms through a vapor–liquid interfacial reaction [32]. The reaction took place between a liquid glycol-soaked ceramic substrate and TiCl_4_ vapor to form a dense skin layer of organometallic hybrid film. The factors of glycols, calcination temperature, and heating atmosphere greatly influenced membrane performance. Ethylene glycol (EG) as a 1,2-diol produces organometallic hybrid materials of titanium ethylene glycolate [Ti-(OCH_2_CH_2_O)_2_] with a two-carbon chain structure. After calcination, molecular-sized membranes with small nanopore sizes are generated, which improve membrane rejection. Propylene glycol as a 1,3-diol forms titanium 1,3-propylene glycolate [Ti-(OCH_2_CH_2_CH_2_O)_2_] with a three-carbon chain structure. The formed CDTO enhances solvent flux due to the formation of large nanopore sizes in nanofilms. Another approach to tuning pore size involves controlling carbon removal. Calcining at higher temperatures and in air atmosphere causes more carbon removal, generating larger pores in CDTO nanofilms. The optimized method of vapor–liquid interfacial reaction provided nanofilms exhibiting a high pure methanol flux of about 125 L·m^−2^·h^−1^·bar^−1^ with a molecular weight cut-off (MWCO) close to 320 Da. These nanofilms afforded long-term stability under harsh conditions due to the rigid nanopores with excellent mechanical and thermal stabilities [32].

In the present study, a nanoporous titanium oxide nanofilm was fabricated by the spin-coating of glycerol onto a flat-sheet α-Al_2_O_3_ ceramic support, followed by an interfacial reaction with TiCl_4_ vapor and a heat treatment process. The OH groups of glycerol, which have structures of both 1,2-diol and 1,3-diol, would undergo crosslinking reactions with TiCl_4_. This process leads to the formation of organometallic hybrid films composed of composites that include two carbon chains from the 1,2-diol and/or three carbon chains from the 1,3-diol. Generally, longer carbon chains can produce larger nanopores, which are beneficial for increasing membrane flux. Shorter carbon chains will generate relatively smaller nanopores, improving solute rejection [31,32]. It is interesting to explore how the unique structure of glycerol affects the performance of pore-size controlled titanium oxide nanofilms. In this study, the effects of vapor–liquid interfacial reaction time and different organic precursors on the separation performance of these membranes were systematically investigated. Finally, the durability and sustained separation efficacy of these membranes were investigated in a methanol solution environment.

## 2. Materials and Methods

### 2.1. Materials and Chemicals

Titanium tetrachloride (TiCl_4_), glycerol (GL), ethylene glycol (EG), and trimethylol propane ethylene (TMP) as precursors were obtained from FuChen Chemical Reagents Factory (Tianjin, China). The dye solutes including Azobenzene, Methyl Orange, Rhodamine B, Indigo Carmine, Congo Red, Rose Bengal, and Picrosirius Red were purchased from the Aladdin Industry Co., Ltd. (Shanghai, China). The flat-sheet α-Al_2_O_3_ ceramic substrate was obtained from Jiangxi Guoci Environmental Protection Technology Co., Ltd., Pingxiang, China, and it was taken as support for nanofilms.

### 2.2. Preparation of GLTO Membranes via Interfacial Reaction

In the typical preparation procedure, as shown in Figure S1, commercial α-Al_2_O_3_ ceramic substrates are first pre-coated with three layers of titanium dioxide (TiO_2_) with nanoparticles of 25 nm, 15 nm, and 5–10 nm to create ultrafiltration ceramic membranes with flat surfaces [14,33]. The flat-sheet ceramic support is then dried in an oven at 80 °C for 2 h to remove any residual moisture. Next, glycerol is deposited on the ceramic support, which has an effective area of about 4 cm^2^, through spin coating at 2000 revolutions per minute (rpm) for 30 s. The carrier coated with glycerol is placed in a closed container with 2 milliliters (mL) of TiCl_4_, which has been preheated to 140 °C. The vapor from the heated TiCl_4_ reacts with the glycerol at the interface, generating an organic–inorganic hybrid film coating on the support (Figure S2). After a certain reaction time, the film is washed with toluene and distilled water and then dried in an oven at 80 °C for 8 h. The carbon-doped TiO_2_ nanofilm, named GLTO, is finally generated by calcination in air in a muffle furnace at 250 °C for 2 h, with a heating rate of 2 °C per minute. This synthesizing procedure results in the formation of a porous carbon-doped titanium oxide nanofilm. The specific characteristics of the nanofilms can be tailored by adjusting the reaction time and the choice of organic precursors.

### 2.3. Membrane Characterization

Field emission scanning electron microscopy (SEM, Zeiss, Oberkochen, Germany) was used to characterize the surface and sectional morphology of each sample. Elemental composition was analyzed by X-ray photoelectron spectroscopy (XPS). Detailed scans of individual elements were used to determine the chemical environment of the atoms, and the area under these curves was used to analyze the elemental composition of the nanofilm. Thermogravimetric analysis (TGA) was performed on a TA instruments Q500 (TA instruments, New Castle, DE, USA). Before starting to heat up, the sample was dried overnight in a 100 °C oven. The temperature increased from 30 to 750 °C at a rate of 10 °C min^−1^. Fourier transform infrared spectroscopy was carried out on a Perkine Elmer 782 Fourier transform spectrophotometer. It was performed between 4000 cm^−1^ and 500 cm^−1^. X-ray diffraction (XRD) was recorded on a Rigaku Ultima IV X-ray Diffractometer (Japan). Diffraction was measured between 2θ = 5° and 80°. The water contact angle was measured at room temperature with a contact angle goniometer. A 3 µL water drop was placed on the surface of the membrane, and the instrument immediately captured an image of the drop. All samples were dried overnight at 80 °C prior to contact angle measurement.

### 2.4. Measurements of Membrane Performances

The performance of the GLTO membrane was evaluated using a dead-end pressure filter with an effective area of 1.3575 cm^2^. Prior to the test, all membranes were pretreated at 3 bar for half an hour to achieve stable penetration values. Then, all separation performance tests were performed at 2 bar. The low concentration of the dye is 20 mg/L, and the high concentration of the dye is 50 mg/L, using a magnetic stirrer to eliminate the concentration polarization effect. To determine the molecular weight cut-offs (MWCOs) of the membranes, their performance in rejecting dyes from methanol solutions was evaluated. The dye concentration was measured by a UV-vis spectrophotometer. In this study, solvent permeability (*P*) and dye retention (*R*) were calculated according to the following Equations (1) and (2), respectively:

(1) $$P=\frac{V}{A\times t\times ∆p}$$

(2) $$R=\left(1-\frac{C_{p}}{C_{f}}\right)\times 100\%$$

where *V* is the volume of the permeating solvent (L), A is the effective area of the permeating membrane (m^2^), t is the permeating time (h), Δ*p* is the transmembrane pressure (bar), *C_p_* is the concentration of dye in the permeating solution (mg/L), and *C_f_* is the concentration of dye in the feeding solution (mg/L).

The pore size distribution of the GLTO nanofilm was determined under the assumption that there were no hydrodynamic interactions between the solutes and the membrane material [32,34]. The dyes in organic solution can be used as model solute molecules. The MWCO value was considered as the 90% rejection of the solute molecular weight for the membrane. Based on the hypothesis, the pore size distribution of the membrane can be expressed as the probability density function, and given as Equation (3):

(3) $$\frac{dR\left(r_{p}\right)}{dr_{p}}=\frac{1}{r_{p}\ln\sigma_{s}\sqrt{2\pi }}e^{\left(-\frac{{(\ln r_{p}-ln\mu_{s})}^{2}}{{2\left(\ln\sigma_{s}\right)}^{2}}\right)}$$

(4) $$\ln r_{p}=-1.4962+0.4654\ln M$$

where *r*_p_ is the Stokes radii of the organic solutes, which can be calculated from Equation (4), *μ*_s_ is the radius of the solute, which gives 50% rejection, *r*_s_ is the size of the solute for which rejection is 84.13%, *σ*_s_ is the geometric standard deviation, which was assumed to be the ratio of the Stokes radius at 84.13% of *R* to the Stokes radius at 50% of *R*, and *M* is the molecular weight of the organic solutes.

### 2.5. Long-Term Stability and Solvent Resistance

In this study, 20 mg/L Congo Red dye was dissolved in methanol, ethanol, and DMF to evaluate the solvent resistance of the GLTO nanofilms. In order to test the long-term stability of the membrane, a 50 mg/L Congo Red methanol solution was used for the 108 h periodic transmembrane filtration test. The filtrate was collected regularly at different times, and the dye concentration was measured by a UV-vis spectrophotometer.

## 3. Results

### 3.1. Characterizations of Membranes

**Figure 1:**
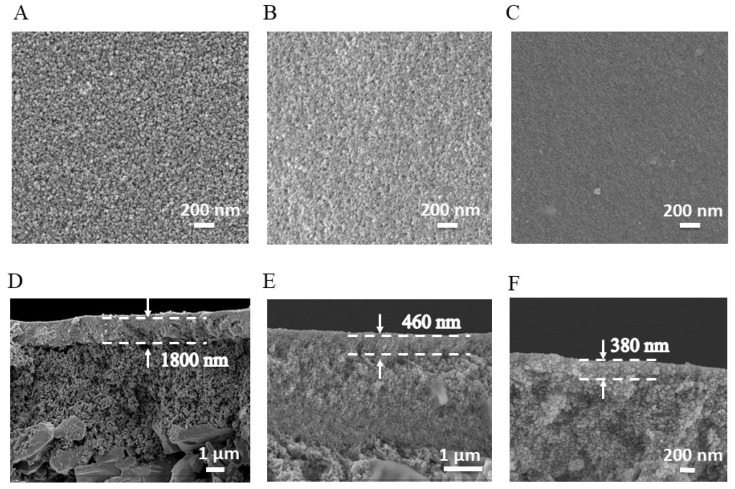
SEM images of samples. (**A**,**D**) surface and cross-section of ceramic substrate; (**B**,**E**) surface and cross-section of organometallic hybrid film after 5 min of reaction; (**C**,**F**) surface and cross-section of GLTO nanofilm after 5 min of reaction and 250 °C calcination.

Glycerol was deposited onto a ceramic substrate through spin coating. Organometallic hybrid films were then generated by reacting TiCl_4_ vapor with glycerol at the vapor–liquid interface for 1 to 6 min. Following calcination in air, carbon-doped TiO_2_ nanofilms derived from glycerol, named GLTO, were synthesized. The surface and cross-sectional morphologies of both the organometallic hybrid films and the resultant GLTO nanofilms were examined using field emission scanning electron microscopy (Figure 1 and Figure S3). Both the organometallic hybrid films and the GLTO nanofilms possessed smooth surfaces after 5 min of interface reaction. Cross-sectional FE-SEM images revealed that the GLTO membrane exhibited a composite structure, featuring a thin, dense functional layer atop a porous ceramic support, as clearly shown in Figure 1. The thickness of the GLTO nanofilms decreased slightly from 460 nm to 380 nm after the thermal treatment of the organometallic hybrid films at 250 °C. Additionally, reducing the reaction time to 3 min further decreased the thickness of the selective layer to 100 nm (Figure S4).

**Figure 2:**
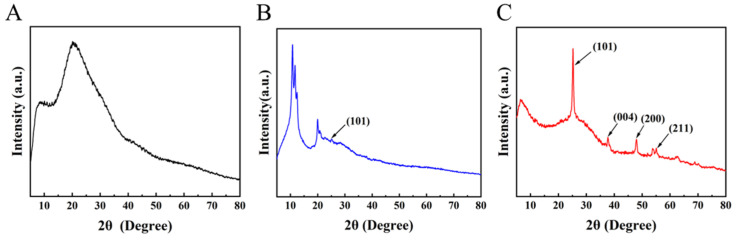
X-ray diffraction (XRD) pattern of samples. (**A**) organometallic hybrid film; (**B**,**C**) GLTO nanofilms thermally treated at 250 °C and 300 °C.

Figure 2 presents the results of an X-ray diffraction (XRD) examination for organometallic hybrid and GLTO samples subjected to thermal treatment at 250 °C and 300 °C under air. A broad peak pattern indicates that the organometallic hybrid sample had an amorphous structure. After calcination, the XRD patterns of GLTO at 250 °C exhibited different peaks, with a small peak at 25.4° corresponding to the diffractions of the (101) crystal planes of the anatase phase in TiO_2_ (JCPDS file No. 21-1272) [35]. When the temperature of thermal treatment was increased to 300 °C, distinct crystalline TiO_2_ peaks appeared. As shown in Figure 2C, the diffraction peaks of the GLTO-300 sample showed significant broadening due to the relatively small size of the TiO_2_ crystallites in GLTO samples [36]. The formation of crystalline TiO_2_ could lead to inter-crystalline defects when two adjacent growing crystals fail to merge together, resulting in large pores and poor rejection [30]. In this case, the thermal treatment temperature should not exceed 300 °C, as it is easier for amorphous materials to form thin films without the possibility of defects and large pores.

**Figure 3:**
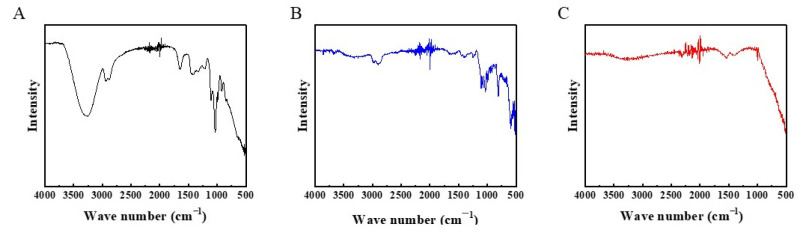
FTIR spectra of samples. (**A**) organometallic hybrid film; (**B**,**C**) GLTO nanofilms thermally treated at 250 °C and 300 °C.

FTIR was employed to further confirm the chemical groups presenting in materials, with the spectra shown in Figure 3. The spectrum of organometallic hybrid samples exhibits a broad peak at 3600–3000 cm^−1^, attributed to the vibration of the O-H bonds in glycerol. The peaks at 2868 and 2930 cm^−1^ correspond to C-H vibrations. The broad peak at 500–600 cm^−1^ is due to the vibration of Ti-O bonds formed from TiCl_4_. After calcination at 250 °C under air, the OH group vibration peaks nearly disappear, and the characteristic peaks of GLTO nanofilms at 2868 and 2930 cm^−1^ weaken. This indicates that the OH groups from glycerol are almost completely removed by calcination, leaving only some organic matter in the GLTO, resulting in carbon-doped TiO_2_ formation. Notably, new peaks appearing at 1000–1150 cm^−1^ can be ascribed to the stretching vibration of the C–O bond. These changes indicate the occurrence of oxygen-containing carbon groups via calcination. The vibration of C-H bonds at 2868 and 2930 cm^−1^ almost disappears after calcination at 300 °C, meaning that high calcination temperatures result in low carbon doping. According to the reported literature [32], low carbon doping in TiO_2_ nanofilms can result in large pore sizes.

Table 1 lists the contact angle values of membranes with water (Figure S5). The contact angle values of organometallic hybrid films increased from 50.856° to 95.993° as the interfacial reaction time extended from 1 min to 3 min. Glycerol, rich in OH groups, facilitates the formation of Ti-O bonds in the presence of TiCl_4_ vapor. However, a short reaction time of 1 min left some unreacted OH groups, resulting in hydrophilicity due to hydrogen bonding with water on the film surface. It was found that 5 min is sufficient for forming a layer of organometallic hybrid film through the interfacial reaction, with a slight increase in contact angle. Calcination enhances the hydrophilicity of GLTO nanofilms, as evidenced by a decrease in contact angle values from 98.895° to 68.782°. During calcination, organometallic hybrid films are converted to GLTO nanofilms with the loss of small molecules. A reduction in carbon content with the loss of organic molecules decreases the contact angle value, resulting in a film with a good hydrophilic surface. In addition, surface porosity also has an influence on the water contact angle. An increase in surface porosity and a reduction in the carbon content of the GLTO nanofilms may be attributed to the decrease in water contact angle. Further research is needed in due time.

**Figure 4:**
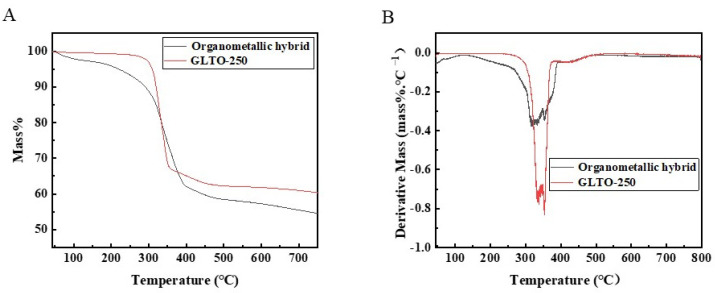
Thermogravimetric analysis of organometallic hybrid and GLTO. (**A**) Mass change with temperature; (**B**) derivative mass change with temperature.

Figure 4 presents the thermogravimetric (TG) curves of organometallic hybrid films and GLTO nanofilms. A gradual mass loss of 11% was observed between 30 °C and 300 °C due to the decomposition of organometallic hybrid materials, resulting in the loss of organic matter. The GLTO sample shows less than 3% mass loss upon heating to 300 °C, with a sharp decrease in weight occurring between 300 °C and 350 °C. This significant loss of molecules led to the formation of the anatase phase in TiO_2_, as seen in the XRD pattern of the GLTO-300 sample with small crystallites. This indicates that porous GLTO nanofilms are stable up to 300 °C in air.

To provide insights into the heating arrangement of organometallic hybrid films, X-ray photoelectron spectroscopy (XPS) was used to report changes in the composition of three different elements: carbon (C), oxygen (O), and titanium (Ti). The results are summarized in Table 2 and Figure S6. The carbon content in the composition increased as the reaction time increased, which is consistent with the results of water contact angle measurements. More carbon doping resulted in a larger water contact angle. It was found that carbon and oxygen had higher elemental contents compared to titanium content, which is attributed to the formation of oxygen-containing carbon groups via calcination, as seen in the FTIR spectroscopy of GLTO nanofilms. Furthermore, when calcination was carried out at 300 °C in air, carbon removal upon heating caused a significant decrease in carbon content. Therefore, surface properties can be adjusted by controlling carbon content through reaction time and heating temperature. Greater hydrophobicity of GLTO nanofilms was achieved with higher carbon doping.

### 3.2. Influence of Reaction Time and Organic Precursors on Membrane Performance

**Figure 5:**
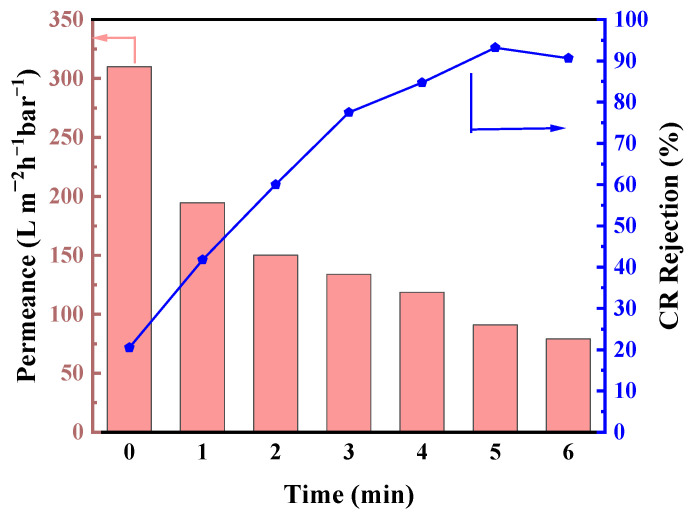
Influence of reaction times on membrane performance of pure methanol flux and CR rejection.

The studies were initiated by the vapor–liquid interface reaction of TiCl_4_ vapor with glycerol under different reaction times, with the results shown in Figure 5. The reaction of TiCl_4_ with glycerol at the vapor–liquid interface for 2 min resulted in a yellow solid film. The synthesized GLTO nanofilm exhibited a pure methanol permeance of 150.1 L·m^−2^·h^−1^·bar^−1^, and the Congo Red (CR) rejection was 50.0%. The rejection reached up to 93.2% with a pure methanol flux of 90.9 L·m^−2^·h^−1^·bar^−1^ when the vapor–liquid interface reaction of TiCl_4_ with glycerol was carried out for 5 min. As the reaction time increased, the methanol permeance gradually decreased and the CR rejection increased, which is due to the thickening of the selective layer in nanofilms with longer reaction times. Considering the favorable dye rejection and high methanol flux simultaneously, a reaction time of 5 min was determined as the most suitable reaction time to prepare GLTO membranes.

**Figure 6:**
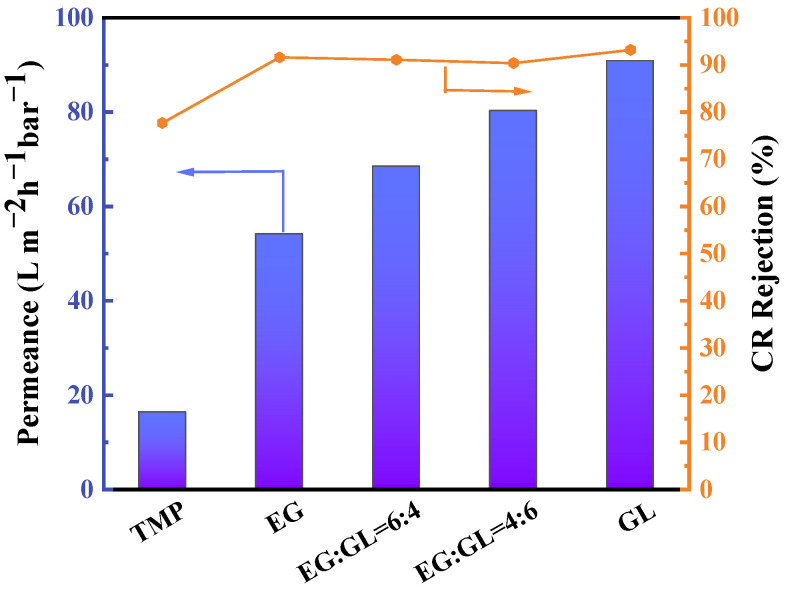
Performance of GLTO membrane prepared from different organic precursors.

The performance of GLTO nanofilms is not only influenced by reaction time but also significantly affected by the choice of organic precursor. A study was conducted to evaluate the permeance of pure methanol and the rejection of Congo Red (CR) by GLTO membranes prepared using different organic precursors: glycerol, ethylene glycol (EG), and trimethylolpropane (TMP). The findings are summarized in Figure 6 and Table S1. When TMP was used as an alternative to glycerol for forming carbon-doped titanium oxide nanofilms, a substantial decrease in methanol permeance was observed, accompanied by a moderate CR rejection rate of 77.7%. TMP’s solid state at room temperature, with a melting point of 58.8 °C, necessitated preheating to 80 °C before deposition on ceramic supports via spin coating. During the vapor–liquid interface reaction with TiCl_4_, TMP tended to crystallize as a solid, resulting in a thick and nonuniform film that compromised performance. In contrast, EG-based membranes exhibited enhanced methanol permeability, reaching 54.1 L·m^−2^·h^−1^·bar^−1^, while maintaining a high solute rejection rate of 91.6%. The combination of EG with glycerol further increased methanol permeance without sacrificing selectivity. The synergy between glycerol and EG as organic precursors has proven to be highly advantageous in achieving both high solvent flux and effective solute rejection. A key factor contributing to the success of glycerol is its unique structure, which features both 1,2-diol and 1,3-diol groups. This distinctive molecular configuration facilitates the creation of pore-size controlled titanium oxide nanofilms during the calcination process, thereby significantly enhancing overall performance. For practical applications in nanofiltration, it is crucial to strike a balance between high permeability and excellent selectivity to achieve optimum results. The ability to finely tune these properties through the selection of organic precursors provides a new approach to membrane separation processes. In the following case, glycerol was used as an organic precursor, and the reaction time was fixed at 5 min, which were considered as optimized preparation parameters for the preparation of GLTO nanofilms.

### 3.3. Separation Performances of GLTO Membranes

**Figure 7:**
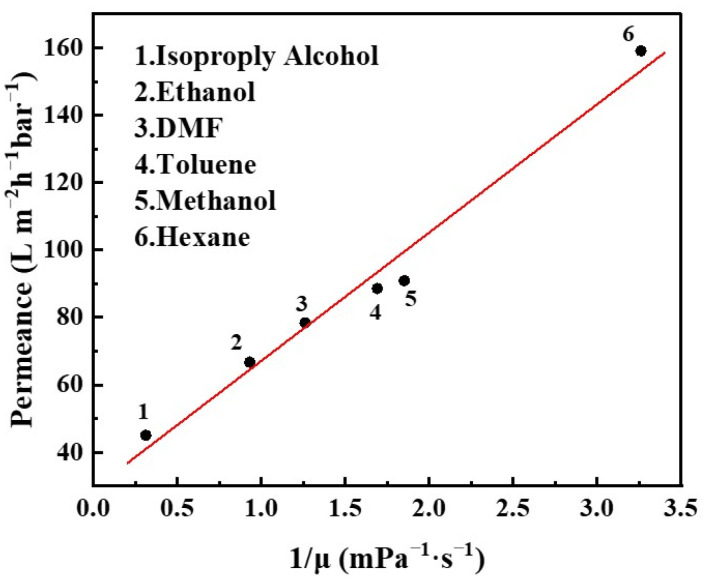
Permeation of various pure solvents through GLTO nanofilm.

The transport properties of various organic solvents through GLTO nanofilms were meticulously examined under a pressure of 0.2 MPa and at room temperature conditions. Remarkably, hexane, the solvent with the lowest viscosity among those tested, exhibited a permeance as high as 159 L·m^−2^·h^−1^·bar^−1^. In contrast, N,N-dimethylformamide (DMF) and ethanol demonstrated solvent fluxes of 78.4 and 66.7 L·m^−2^·h^−1^·bar^−1^, respectively, while the flux values for other solvents are detailed in Figure 7 and Table S2. The empirical data derived from the permeance studies of diverse solvents through GLTO nanofilms revealed a salient trend. The permeance of pure solvents demonstrated an almost direct proportionality to their respective viscosities. This observation strongly implies that the solvent-GLTO nanofilm interactions are notably minimal, suggesting that the permeation process is predominantly influenced by hydrodynamic factors rather than specific intermolecular interactions at the solvent–membrane interface. This finding carries significant implications for the application of GLTO nanofilms in solvent separation technologies. It suggests that the performance efficiency of these membranes, as measured by solvent permeance, can be strategically optimized based on the intrinsic viscosity characteristics of the solvents used. This insight aligns with previous research on other OSN membranes with rigid pores, which also found minimal impact of Hansen solubility parameters on permeance [6,37]. Such understanding facilitates the design and enhancement of separation processes, ensuring operational efficacy and efficiency.

**Figure 8:**
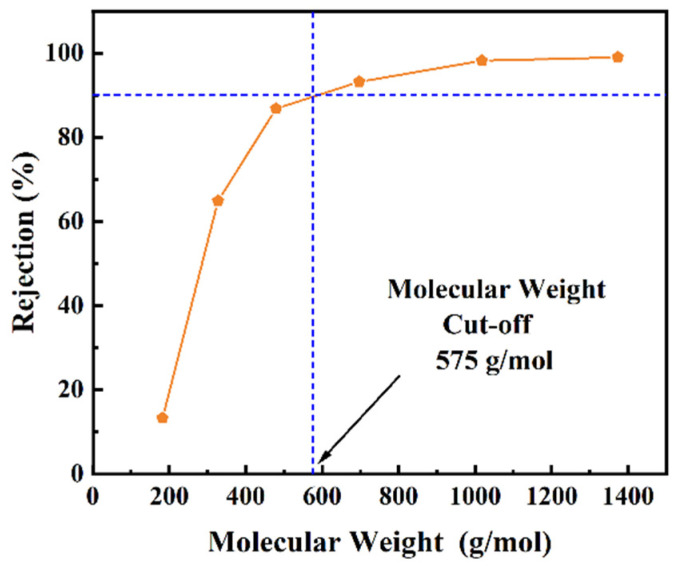
Rejection behaviors of GLTO nanofilm toward various dyes in methanol solution.

To further characterize the performance of these synthesized membranes, molecular weight cut-off (MWCO) measurements were conducted using a dead-end filtration setup. The rejection experiments, carried out in methanol, yielded insightful results depicted in Figure 8 and Table S3. Notably, the rejection rates for Picrosirius Red (1373 Da) and Rose Bengal (1017 Da) through the GLTO membrane reached up to 99%. It became evident that the dye rejection rate increased progressively with the molecular weight of the dye, establishing a direct correlation. Given that the MWCO is defined at a 90% rejection threshold, the determined MWCO value for the GLTO nanofilm approximated 575 Da. This conclusion was substantiated by the molecular weight distribution curve of the feed solute, affirming the distinction in molecular sizes of the dyes. When comparing the rejection of negatively charged Indigo Carmine IC (466.36 Da) and positively charged Rhodamine B (479.01 Da), it was observed that their rejection percentages through the GLTO membrane were nearly identical (Figure S7). This finding underscores that rejection is predominantly governed by molecular size rather than the charge of the dye, reinforcing the notion of a size-selective filtration mechanism [32].

**Figure 9:**
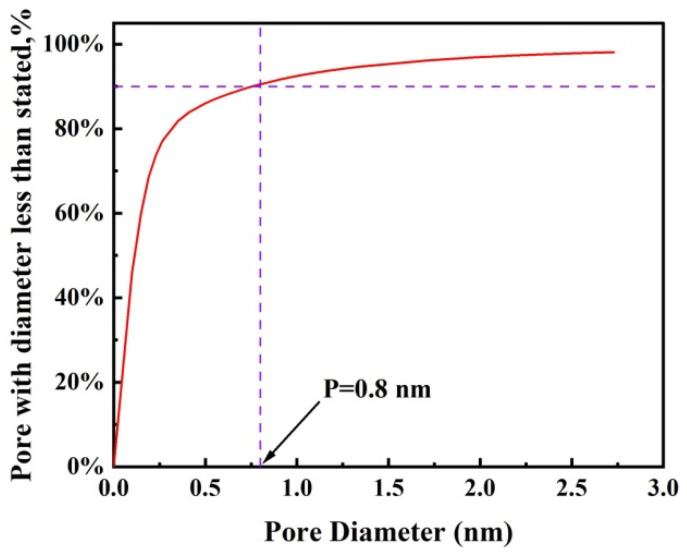
Cumulative pore size distribution curve of the GLTO membrane.

To estimate the pore size and its distribution within the GLTO nanofilms, rejection data collected from the membrane were modeled. Since the charge of the dye did not influence rejection, it was feasible to correlate the molecular weight of the solute directly to the size of the dye molecule. Employing Ferry’s equation facilitated the estimation of the correlation of the MWCO with the pore size of the GLTO membrane [38]. The membrane’s pore size was approximately 0.92 nm corresponding to an MWCO value of 575 Da. Recognizing the necessity to account for a distribution of pore sizes within the GLTO nanofilm, a theoretical model outlined in Equation (3) was utilized, assuming negligible hydrodynamic interactions between the membrane material and organic solutes [32,34]. The pore size distribution and probability density function curve were calculated and indicated in Figure 9. It can be found that about 90% of the pores have a size of less than 0.8 nm. This approach provided a reasonable approximation of the pore size distribution, revealing that the GLTO nanofilms possess limited pore size distributions coupled with efficient transport pathways.

### 3.4. Solvent Resistance and Long-Term Stability of Membranes

**Figure 10:**
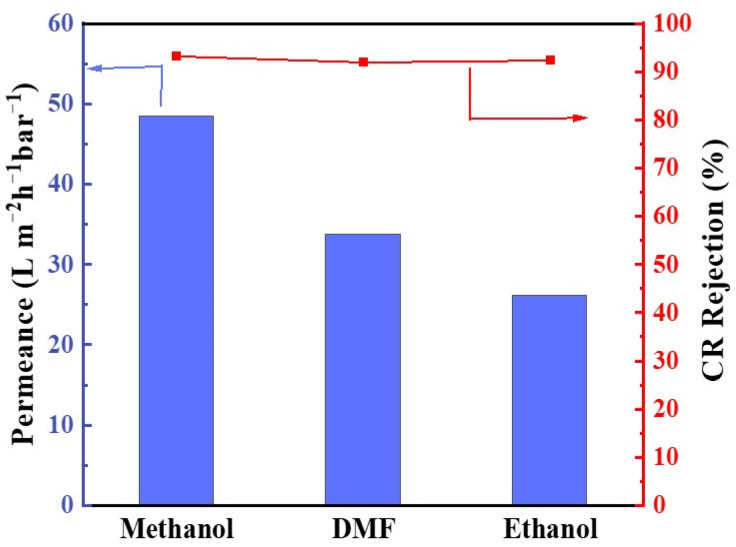
Rejection of Congo Red by the GLTO membrane in different solvents.

The synthesis of fine chemicals, pharmaceuticals, and agrochemicals frequently demands stringent reaction conditions involving aggressive solvents. Membranes exhibiting high stability under such extreme conditions are crucial as they can significantly mitigate energy consumption by efficiently separating organic solvents from the reaction mixture. In this context, GLTO nanofilms were meticulously fabricated under optimized parameters, and their solvent resistance was assessed utilizing a 20 mg L^−1^ Congo Red (CR) solution in methanol, ethanol, and DMF, respectively. The outcomes, depicted in Figure 10, reveal a discernible decline in permeation flux when transitioning from methanol to ethanol and DMF, yet the CR rejection rate remained essentially invariant. This consistent separation efficiency of CR across varying solvents implies that the pores within the GLTO membrane maintain rigidity even in harsh solvent environments, signifying effective solute rejection irrespective of the solvent medium and affirming the superior solvent resistance of the GLTO nanofilm.

**Figure 11:**
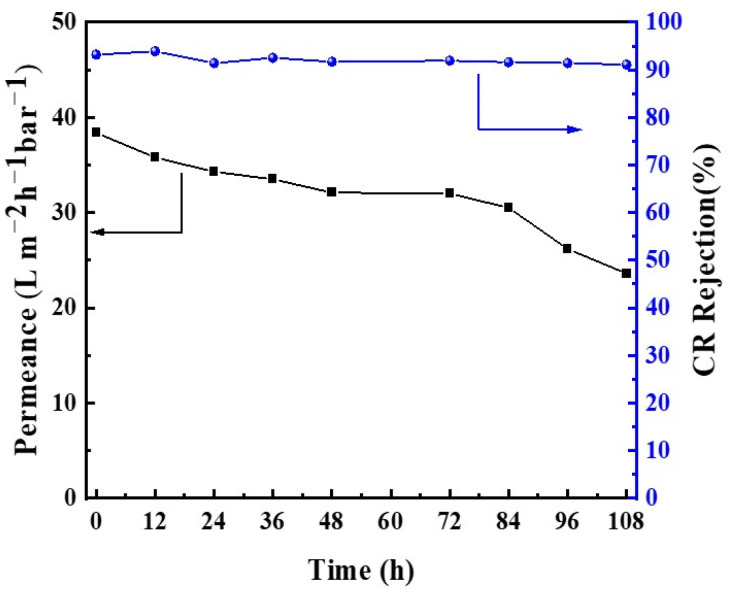
Long-term stability of the GLTO membrane for dye rejection. Test conditions: 0.2 MPa, 20 mg⋅L^−1^ Congo Red methanol solution at room temperature.

To further scrutinize the enduring stability of the GLTO membrane, an exhaustive 108 h transmembrane filtration experiment was conducted employing a 50 mg L^−1^ CR-methanol solution at ambient temperature. The membrane was intermittently immersed in methanol containing dissolved CR and subjected to periodic transmembrane pressure application to collect permeate. As illustrated in Figure 11, the dye rejection rate persisted almost unchanged throughout the duration, demonstrating commendable rejection performance toward CR in methanol. The solvent flux of the membrane decreased gradually with about 23 L·m^−2^·h^−1^·bar^−1^ till the end of the test. This extended 108 h test underscores the long-term efficacy of the OSN membrane synthesized herein, highlighting its potential for sustainable applications in challenging chemical processes.

### 3.5. Performance Comparison with Other OSN Membranes

To comprehensively highlight the superior performance of GLTO membranes, a comparative analysis was undertaken against OSN membranes documented in the prior literature. Table 3 encapsulates the methanol permeance and dye rejection for these benchmarked OSN membranes, establishing a clear comparison framework. It could be seen that the GLTO membrane is effective at removing CR in a methanol solution with a remarkable CR rejection and a high methanol flux. Membrane technology is inherently governed by a trade-off between flux and rejection. Typically, a membrane exhibiting high permeate flux may compromise on selectivity, but the GLTO membrane performed good methanol permeance without sacrificing solute rejection efficacy. Furthermore, this study extends beyond conventional assessments by evaluating the GLTO membrane’s resilience against potent polar solvents like DMF. Despite the challenging nature of DMF, the GLTO membrane retained its high dye rejection prowess, underscoring its suitability for organic solvent systems prevalent in the chemical industry and allied sectors. This robust performance can be attributed to the new fabrication technique involving a vapor–liquid interfacial reaction between glycerol and TiCl_4_ vapor, which paves the way for tailoring nanofilm properties with excellent performance.

## 4. Conclusions

In summary, a porous carbon-doped titanium oxide nanofilm (GLTO) was successfully fabricated through a vapor–liquid interfacial reaction involving glycerol and TiCl_4_ vapor, followed by calcination at 250 °C in air. The surface properties of the nanofilm can be finely tuned by adjusting the carbon content, which is controlled by varying the reaction time and calcination temperature. High carbon content imparts an enhanced hydrophobicity to the GLTO nanofilms. The reaction between glycerol and TiCl_4_ facilitates the formation of crosslinks involving 1,2-diol and/or 1,3-diol groups, leading to an organometallic hybrid structure. Upon calcination, this process generates nanopores with an MWCO value of approximately 575 Da, endowing the GLTO nanofilm with high solvent permeance and effective solute rejection capabilities. Notably, the as-synthesized nanofilm demonstrates exceptional efficacy in removing Congo Red (CR) from methanol solutions, achieving a CR rejection rate of 93.2% while maintaining a high methanol flux. Furthermore, the nanopores within the membrane exhibit remarkable rigidity even in the presence of strong polar solvents like DMF. This unique combination of properties enables the GLTO nanofilm to provide long-term, stable, and efficient organic separation performance under harsh conditions, making it a highly promising material for advanced filtration applications in the chemical industry and related fields.

## Figures and Tables

**Table 1:** Water contact angle of organometallic hybrid and GLTO film at different reaction times.

Sample	Interfacial Reaction Time		
	1 min	3 min	5 min
Organometallic hybrid film	50.856°	95.993°	98.895°
GLTO nanofilm	34.641°	68.039°	68.782°

**Table 2:** Chemical composition of GLTO nanofilms based on atomic percentage.

Interfacial Reaction Time	Calcination Temperature	Carbon (%)	Titanium (%)	Oxygen (%)
1 min	250 °C	52.11	8.96	38.93
3 min	250 °C	62.21	6.61	31.19
5 min	250 °C	63.80	5.87	30.33
5 min	300 °C	30.84	21.74	47.42

**Table 3:** Comparisons of permeances and MWCOs of the GLTO membrane with that of some OSN membranes in the literature.

Membrane	Pure Solvents Permeance (L·m^−2^·h^−1^·bar^−1^)	Dye Used and Its Molecular Weight (g·mol^−1^)	Rejection (%)	Ref.
GLTO	Methanol 90.9	Congo Red (696.7)	93.2	This work
CDTO-Air-30%H_2_O/AAO	Methanol 125	Congo Red (696.7)	99	[32]
TA_0.125_-Ca/FeSA	Methanol 107.7	Congo Red (696.7)	97	[7]
PIM-1/Al_2_O_3_	Methanol 2.0	Congo Red (696.7)	99	[14]
u-Ti_3_C_2_T_x_@P84 MMMsx	Methanol ≈ 7	Congo Red (696.7)	97	[39]
PA/PDA-HKUST-1_0.6_/PEI	Methanol 0.9	Congo Red (696.7)	92	[40]
af-GQDs	Methanol 13.5	Rhodamine B (479)	99	[41]

## Data Availability

The original contributions presented in the study are included in the article and Supplementary Materials, further inquiries can be directed to the corresponding authors.

## Supplementary Materials

The following supporting information can be downloaded at: https://www.mdpi.com/article/10.3390/membranes14110233/s1. Figure S1. Schematic of the preparation procedure of GLTO nanofilms on porous ceramic support; Figure S2. Reaction for the formation of organometallic hybrid material; Figure S3. SEM images of samples. (A) surface of GLTO nanofilm after 2 min of reaction; (B) surface of GLTO nanofilm after 3 min of reaction; (C) surface of GLTO nanofilm after 4 min of reaction; (D) surface of GLTO nanofilm after 6 min of reaction; Figure S4. SEM images of samples. (A) cross-section of organometallic hybrid film after 3 min of reaction; (B,C) cross-section of GLTO nanofilm after 3 min of reaction and image enlargement; Figure S5. The surface water contact angle of the samples. (A) organometallic hybrid at 1 min of reaction; (B) organometallic hybrid at 3 min of reaction; (C) organometallic hybrid at 5 min of reaction; (D) GLTO nanofilm at 1 min; (E) GLTO nanofilm at 3 min; (F) GLTO nanofilm at 5 min; Figure S6. Chemical composition analysis via XPS. XPS full scan (A) GLTO nanofilm at 1 min and calcined at 250 °C, (B) GLTO nanofilm at 3 min and calcined at 250 °C, (C) GLTO nanofilm at 5 min and calcined at 250 °C, and (D) GLTO nanofilm at 5 min and calcined at 300 °C; (E) C1s deconvolution of GLTO nanofilm at 5 min and calcined at 250 °C, (F) GLTO nanofilm at 5 min and calcined at 300 °C; Figure S7. GLTO membrane performance based on size and charge of dyes. Rhodamine B as positive dye and Indigo Carmine as negative dye; Table S1: Performance of GLTO membrane prepared from different organic precursors. Table S2: Permeation of pure solvents through GLTO nanofilm; Table S3: Rejection behaviors of GLTO nanofilm toward various dyes in methanol solution.
