# Supplementary material for: Carbon-Doped TiO2 Nanofiltration Membranes Prepared by Interfacial Reaction of Glycerol with TiCl4 Vapor
Source: Membranes (Basel). 2024 Nov 7;14(11):233. doi: 10.3390/membranes14110233 (PMC11596831; doi:10.3390/membranes14110233)
Supplement: Supplementary file 1 [file membranes-14-00233-s001.zip › membranes-3294602-supplementary.pdf]

Supporting Information

Carbon-doped TiO_2 nanofiltration membranes prepared by interfacial reaction of glycerol with $TiCl_4$ vapor

Wenjing Zhang, Jiangzhou Luo, Honglei Ling, Lei Huang and Song Xue**

School of Chemistry & Chemical Engineering, Tianjin Key Laboratory of Organic Solar Cells and Photochemical Conversion, Tianjin University of Technology, Tianjin, 300384, China

* Corresponding authors: luojz@email.tjut.edu.cn; xuesong@ustc.edu.cn

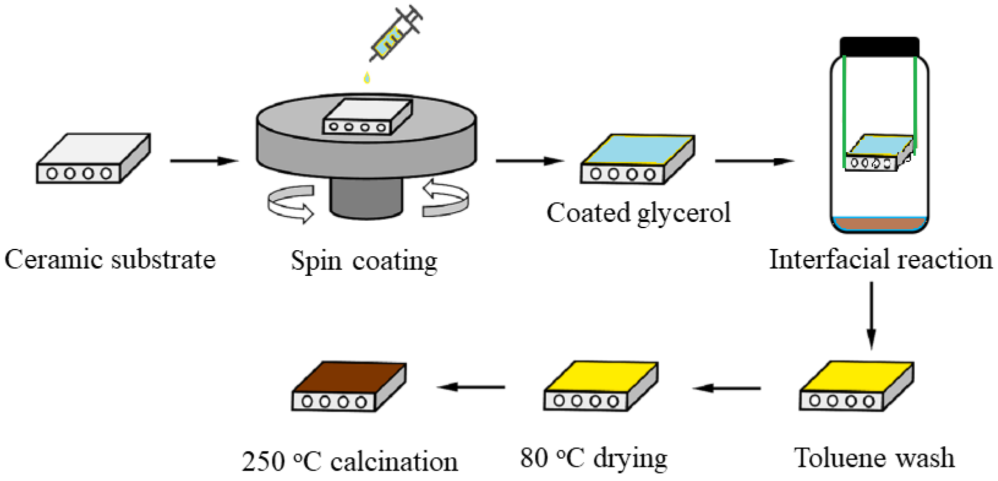

Fig. S1. Schematic of the preparation procedure of GLTO nanofilms on porous ceramic support.

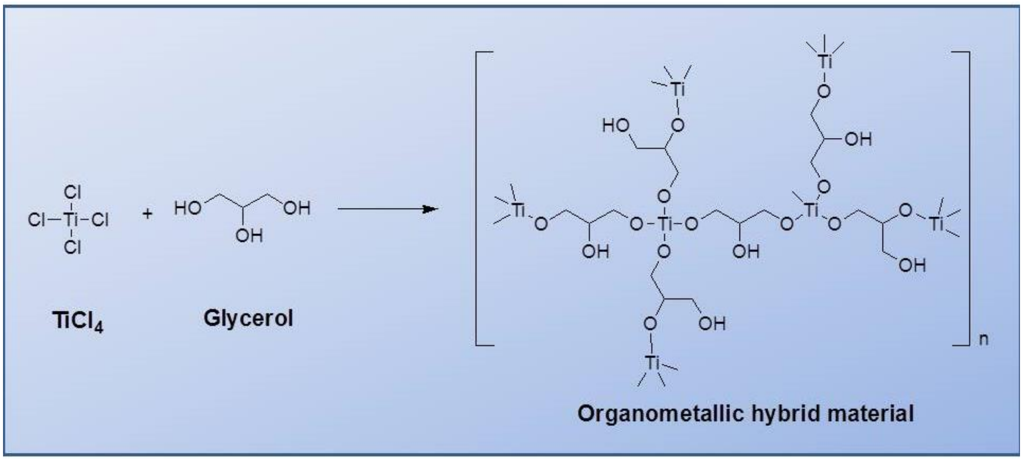

Fig. S2. Reaction for the formation of organometallic hybrid material

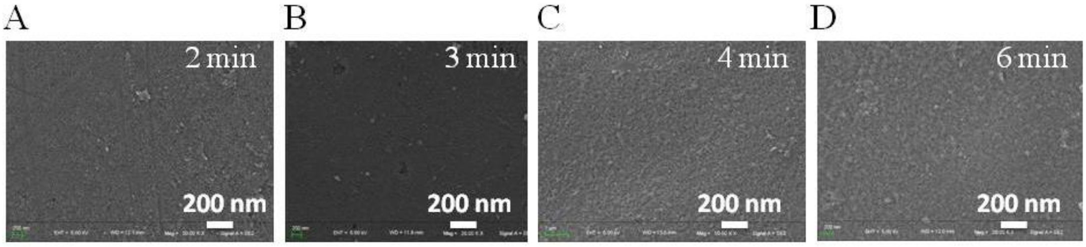

Fig. S3. SEM images of samples. (A) surface of GLTO nanofilm after 2 min of reaction; (B) surface of GLTO nanofilm after 3 min of reaction; (C) surface of GLTO nanofilm after 4 min of reaction; (D) surface of GLTO nanofilm after 6 min of reaction; The surface of GLTO nanofilm after 5 min of reaction was shown in text.

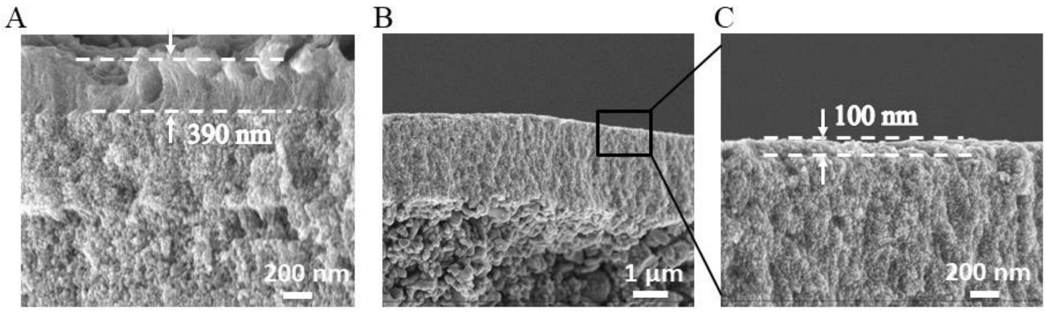

Fig. S4. SEM images of samples. (A) cross-section of organometallic hybrid film after 3 min of reaction; (B,C) cross-section of GLTO nanofilm after 3 min of reaction and image enlargement.

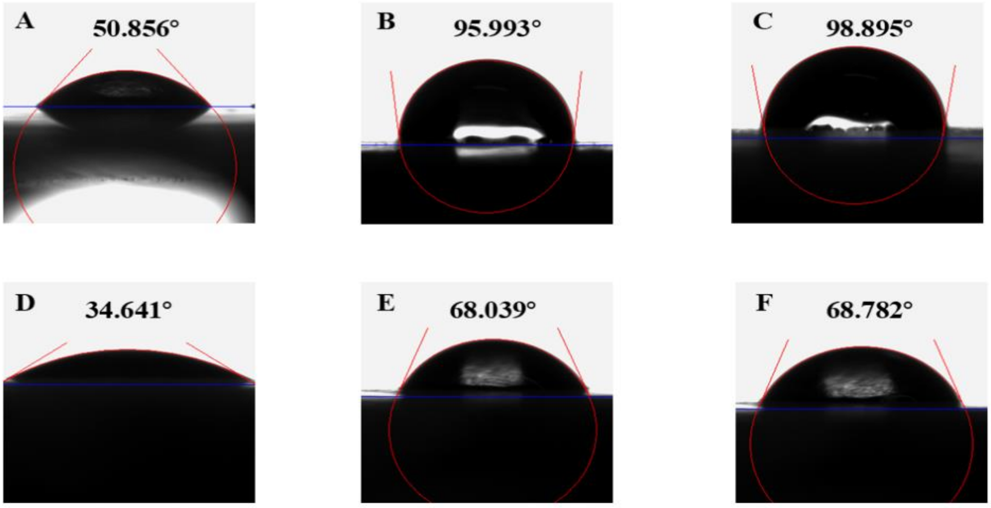

Figure S5. The surface water contact angle of the samples. (A) organometallic hybrid at 1 min of reaction; (B) organometallic hybrid at 3 min of reaction; (C) organometallic hybrid at 5 min of reaction; (D) GLTO nanofilm at 1 min; (E) GLTO nanofilm at 3 min; (F) GLTO nanofilm at 5 min.

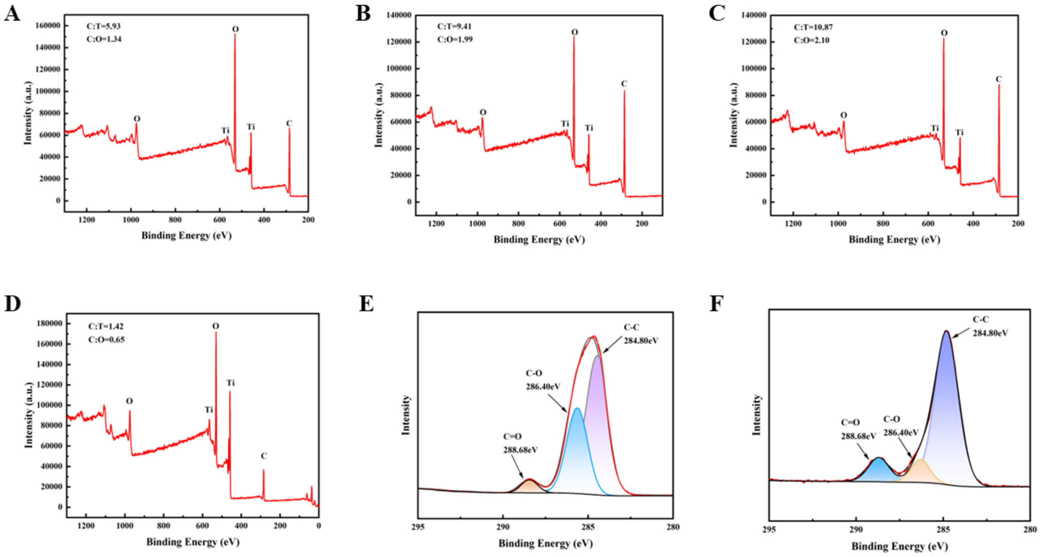

Fig. S6. Chemical composition analysis via XPS. XPS full scan (A) GLTO nanofilm at 1 min and calcined at 250 °C, (B) GLTO nanofilm at 3 min and calcined at 250 °C, (C) GLTO nanofilm at 5 min and calcined at 250 °C, and (D) GLTO nanofilm at 5 min and calcined at 300 °C; (E) C1s deconvolution of GLTO nanofilm at 5 min and calcined at 250 °C, (F) GLTO nanofilm at 5 min and calcined at 300 °C.

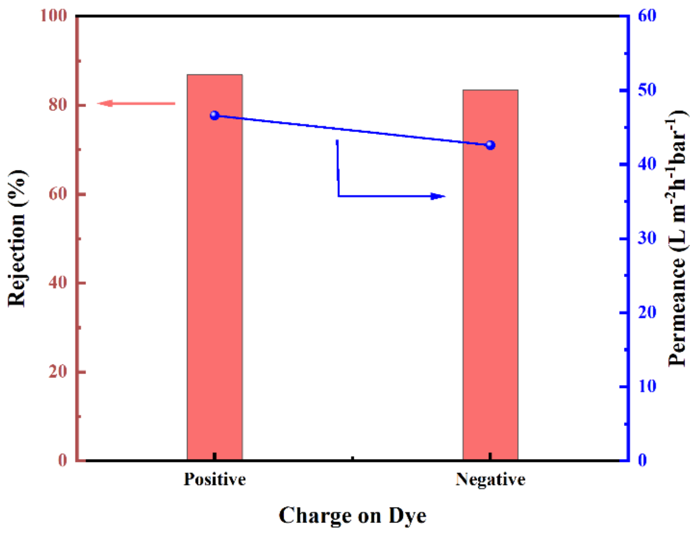

Fig. S7. GLTO membrane performance based on size and charge of dyes. Rhodamine B as positive dye and Indigo Carmine as negative dye.

Table S1. Performance of GLTO membrane prepared from different organic precursors.

Organic precursor	Permeance (L $m^{-2}h^{-1}bar^{-1}$)	CR Rejection (%)
TMP	16.3	77.7
EG	54.1	91.6
EG:GL=6:4	68.5	91.1
EG:GL=4:6	80.3	90.4
GL	90.9	93.2

Table S2. Permeation of pure solvents through GLTO nanofilm

	$1/\mu$ ($mPa^{-1}\cdot s^{-1}$)	Permeance ($L\ m^{-2}h^{-1}bar^{-1}$)
Isoproply alcohol	0.31	45.0
Ethanol	0.93	66.7
DMF	1.26	78.4
Toluene	1.69	88.6
Methanol	1.85	90.9
Hexane	3.26	159.1

Table S3. Rejection behaviors of GLTO nanofilm toward various dyes in methanol solution

Dye	Electric charge	Molecular Weight (g/mol)	Rejection (%)
Azobenzene	-	182.22	13.3
Methyl Orange	-	327.33	64.9
Indigo Carmine IC	-	466.36	83.5
Rhodamine B	+	479.01	86.8
Congo Red	+	696.68	93.2
Rose Bengal	-	1017.63	98.2
Picrosirius Red	-	1373.05	99.0
